# Lack of a Clear Behavioral Phenotype in an Inducible FXTAS Mouse Model Despite the Presence of Neuronal FMRpolyG-Positive Aggregates

**DOI:** 10.3389/fmolb.2020.599101

**Published:** 2020-12-14

**Authors:** Saif N. Haify, Ruchira S. D. Mankoe, Valerie Boumeester, Esmay C. van der Toorn, Rob F. M. Verhagen, Rob Willemsen, Renate K. Hukema, Laurens W. J. Bosman

**Affiliations:** ^1^Department of Clinical Genetics, Erasmus MC, Rotterdam, Netherlands; ^2^Department of Neuroscience, Erasmus MC, Rotterdam, Netherlands; ^3^Department of Health Care Studies, Rotterdam University of Applied Sciences, Rotterdam, Netherlands

**Keywords:** FXTAS, nuclear inclusions, mouse behavior, *FMR1*, repeat expansion

## Abstract

Fragile X-associated tremor/ataxia syndrome (FXTAS) is a rare neurodegenerative disorder caused by a 55–200 CGG repeat expansion in the 5′ untranslated region of the Fragile X Mental Retardation 1 (*FMR1*) gene. FXTAS is characterized by progressive cerebellar ataxia, Parkinsonism, intention tremors and cognitive decline. The main neuropathological hallmark of FXTAS is the presence of ubiquitin-positive intranuclear inclusions in neurons and astrocytes throughout the brain. The molecular pathology of FXTAS involves the presence of 2 to 8-fold elevated levels of *FMR1* mRNA, and of a repeat-associated non-AUG (RAN) translated polyglycine peptide (FMRpolyG). Increased levels of *FMR1* mRNA containing an expanded CGG repeat can result in cellular toxicity by an RNA gain-of-function mechanism. The increased levels of CGG repeat-expanded *FMR1* transcripts may create RNA foci that sequester important cellular proteins, including RNA-binding proteins and FMRpolyG, in intranuclear inclusions. To date, it is unclear whether the FMRpolyG-positive intranuclear inclusions are a cause or a consequence of FXTAS disease pathology. In this report we studied the relation between the presence of neuronal intranuclear inclusions and behavioral deficits using an inducible mouse model for FXTAS. Neuronal intranuclear inclusions were observed 4 weeks after dox-induction. After 12 weeks, high numbers of FMRpolyG-positive intranuclear inclusions could be detected in the hippocampus and striatum, but no clear signs of behavioral deficits related to these specific brain regions were found. In conclusion, the observations in our inducible mouse model for FXTAS suggest a lack of correlation between the presence of intranuclear FMRpolyG-positive aggregates in brain regions and specific behavioral phenotypes.

## Introduction

Fragile X-associated tremor/ataxia syndrome (FXTAS) is a late-onset neurodegenerative disease that is characterized mainly by essential tremor, cerebellar ataxia, Parkinsonism, peripheral neuropathy and cognitive decline (Hagerman et al., 2001; Tassone et al., 2007; Hagerman and Hagerman, 2013, 2016). FXTAS leads to cerebral and cerebellar atrophy, with increased T2 signal intensity in MRI images of the middle cerebellar peduncles as diagnostic hallmark (Brown and Stanfield, 2015). Carriers of a premutation in the *FMR1* gene, consisting of a 55–200 CGG repeat expansion, are at risk of developing FXTAS. Such intermediate repeat expansions lead to elevated levels of *FMR1* mRNA (Tassone et al., 2000; Kenneson et al., 2001; Salcedo-Arellano et al., 2020). In contrast, longer repeat expansions, more than 200 units, induce silencing of *FMR1* mRNA, which results in a lack of FMRP protein, causing the neurodevelopmental Fragile X syndrome (Bassell and Warren, 2008; Salcedo-Arellano et al., 2020).

Several mechanisms by which the premutation and the consequential increase in *FMR1* mRNA levels may lead to the development of FXTAS have been proposed. Of these, arguably the most studied process is the formation of intranuclear inclusions that has been very well-documented in patients as well as in animal models and their occurrence has been linked to alterations at the cellular level in neurons and astrocytes (Louis et al., 2006; Jin et al., 2007; Berman et al., 2014; Ma et al., 2019; Haify et al., 2020). The intranuclear inclusions are mainly composed of proteins and to date more than 200 different proteins have been identified in nuclear inclusions (Iwahashi et al., 2006; Ma et al., 2019). *FMR1* mRNA containing a CGG repeat expansion, although present itself only in relatively low concentrations in the nuclear inclusions, could act as a scaffold binding place for the other components (Cid-Samper et al., 2018; Langdon et al., 2018; Ma et al., 2019). The putative pathogenicity of these inclusions could be based on depleting essential molecules, including RNA-binding proteins (Jin et al., 2007; Sofola et al., 2007; Qurashi et al., 2011; Sellier et al., 2013). Another, not necessarily mutually exclusive, potential pathogenic mechanism is repeat-associated non-AUG (RAN) translation through which a toxic polyglycine (FMRpolyG) protein is produced from the elongated *FMR1* CGG repeat mRNA (Todd et al., 2013; Sellier et al., 2017; Krans et al., 2019). To date, the relative contributions of the RNA-based inclusions and the expression of toxic FMRpolyG to human pathology are still matter of debate. It has even been suggested that in early disease state, the inclusions may serve a protective function by sequestering FMRpolyG (Greco et al., 2006; Hagerman and Hagerman, 2016).

Our current clinical, molecular and histopathological understanding of FXTAS in patients is mostly derived from studies in mouse models. Several mouse models have been generated to study the (neuro)pathology and behavioral effects of FXTAS. Initially two knock-in (KI) mouse models were generated: the Dutch (CGG_*dut*_) and the NIH (CGG_*nih*_) KI mouse model. Both KI mouse models display FXTAS pathology at the genetic, molecular, histological and behavioral level with slight differences. Both show ubiquitin-positive intranuclear inclusions throughout the entire brain, but these inclusions are more common in the CGG_*dut*_ KI mice. Behavioral examination of both CGG KI mice revealed memory impairment (Hunsaker et al., 2009), increased levels of anxiety in the CGG_*dut*_ KI mice while CGG_*nih*_ KI mice show decreased levels of anxiety. Also, assessment of motor function in the CGG_*dut*_ KI mouse model showed impairment with increasing age of the mice (Van Dam et al., 2005). This observed cognitive decline and motor function impairment in these mice may reflect the progressive cognitive decline and functionality impairment observed in FXTAS patients. Although both KI mouse models nicely recapitulate FXTAS disease pathology, the time to generate a phenotype is a major disadvantage. It takes roughly up to 52–72 weeks before any phenotype is observed in these mice. Therefore, several transgenic mouse models were developed to study specific research questions of FXTAS disease pathology such as RAN-translation, mRNA containing expanded CGG repeat and potential therapeutic interventions. We refer the reader to more advanced and detailed reviews covering all available mouse models for the premutation and FXTAS (Berman et al., 2014; Haify et al., 2020). All these mouse models show presence of ubiquitin-positive and FMRpolyG-positive inclusions in the central nervous system (CNS) in neurons and astrocytes as well as in non-CNS organs, thus display the most prominent neuropathological hallmark in FXTAS disease pathology, with the notable exception of the intention tremor.

We studied the occurrence of intranuclear inclusions in a novel inducible mouse model for FXTAS, and related these to quantitative alterations in mouse behavior. To avoid interactions during development, we induced—in adult mice—the expression of a randomly integrated 103× CGG repeat expansion in the mouse under control of the neuron-specific Ca^2+^/calmodulin-dependent protein kinase II alpha (*CamKII-*α) promoter. The *CamKII-*α driver induces expression throughout the entire forebrain, but also in several other regions in the cerebrum such as the hippocampus and the basal ganglia, which are regions known to be involved in FXTAS disease pathology (Greco et al., 2002; Wang et al., 2013). In this report we mainly focused on the dentate gyrus (DG) and CA3 region of the hippocampus, and the striatum, being part of the basal ganglia. These regions are believed to contribute to several behavioral impairments in FXTAS such as in motor learning and coordination, and memory (Scaglione et al., 2008; Hagerman and Hagerman, 2013; Haify et al., 2020). Also, cognitive decline based on performance in spatial learning, memory tasks, executive motor function impairments and anxiety associated disorders are observed in premutation carriers and FXTAS patients (Hasegawa et al., 2009). For a period of 3 months after induction, we quantified the formation of inclusions in the brain and characterized the behavioral performance. As expected, and in line with the expression pattern of the *CamKII-*α promoter (Burgin et al., 1990), we found intranuclear inclusions in the hippocampus and the striatum, already appearing 4 weeks after dox-induction. To our surprise, however, virtually no impact on behavioral performance was detectable even after 3 months of dox-induction. We therefore propose based on this study that intranuclear inclusions do not have an immediate detrimental effect on neuronal function and this may point to a protective function of inclusion formation in the early-onset of disease-progression in FXTAS.

## Materials and Methods

### Mice

**FIGURE 1:**
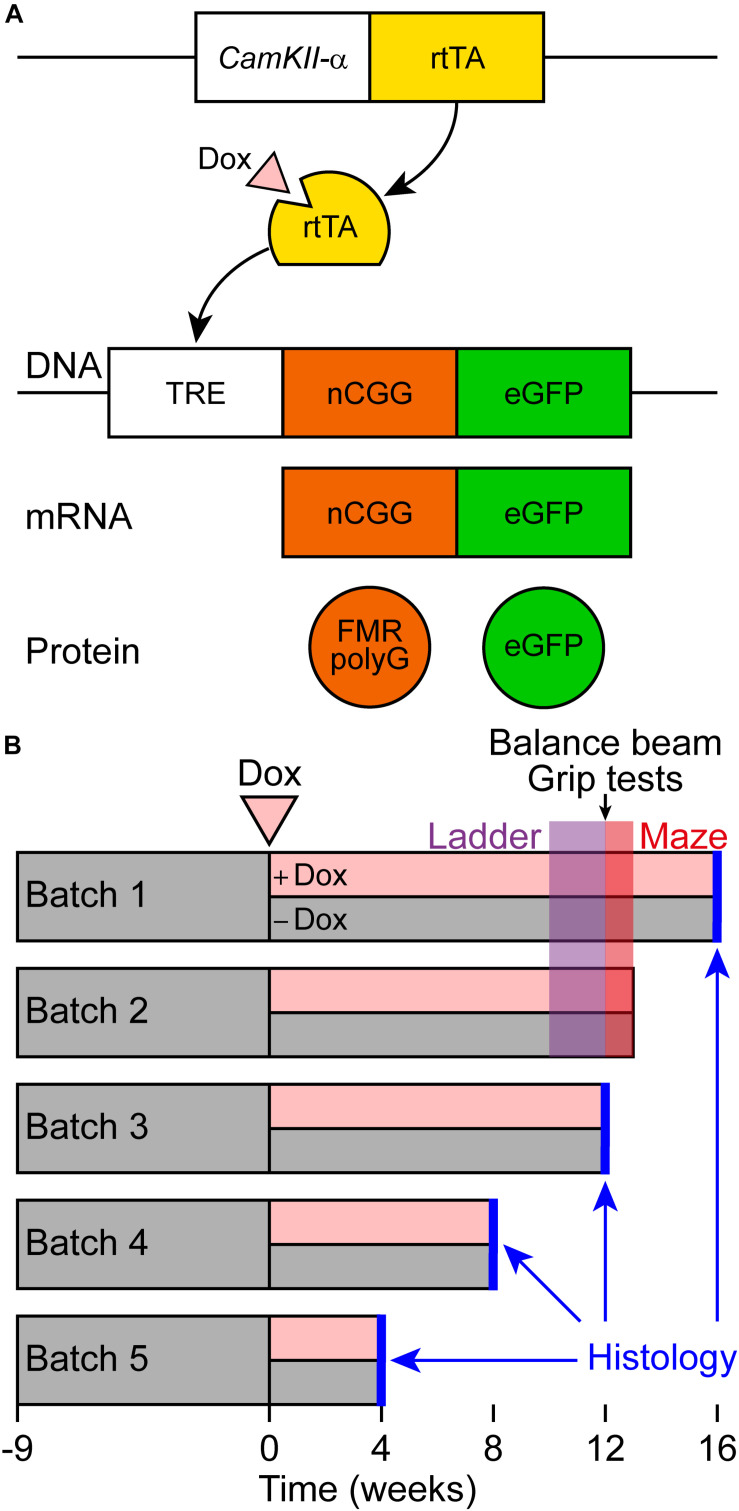
Schematic representation of the Tet-On system and behavioral testing of a new brain-specific mouse model for FXTAS. **(A)** Brain-specific expression of the expanded CGG repeat RNA coupled to GFP was studied in the *CamKII-α-*rtTA/TRE-103CGG-GFP inducible mouse model with a C57BL6/J background. The Tet-On system was used to generate double transgenic mice expressing the expanded CGG repeat at the RNA level. Expression of the reverse tetracycline transactivator (rtTA) is controlled by the *CamKII-*α promoter on a separate transgene. Upon dox administration, rtTA will be activated and can bind the tet response element (TRE) on another transgene, which induces expression of the expanded CGG repeat at the RNA level and GFP at the protein level. As the transgene contains the 5’-UTR of the FMR1 gene with an expanded CGG repeat, the FMRpolyG polypeptide is produced from the expanded CGG repeat by RAN translation. **(B)** Schematic overview of the experimental schedule for histological analysis and behavioral testing. At around 9 weeks of age, dox-treatment started. Around 10 weeks later, ErasmusLadder tests were performed, followed by balance beam and grip tests. Finally, the mice were subjected to the Morris water maze test.

For this study, male and female *CamKII-*α-rtTA/TRE-103CGG-GFP-mice with a C57BL6/J background were used (Figure 1A). This *CamKII-*α inducible mouse model was generated similarly to the ubiquitous inducible mouse model by random integration of the transgenes in the genome (Hukema et al., 2014). The TRE-103CGG-GFP mice were crossed with the *CamKII-*α-rtTA driver line to generate double transgenic mice using the Tet-On system. Dox-treatment was initiated at the age of 9 weeks in these mice. Dox drinking water contained 2 mg/ml doxycycline hyclate (Sigma) in 5% sucrose (Sigma) and was refreshed every 2–3 days.

### Genotyping

For genotyping, toe clips from P5–7 mice and, after sacrificing, lung tissue, were incubated overnight in 300 μl tail digestion buffer [TDB; 50 mM KCl, 10 mM Tris-HCl pH 9, 0.1% Triton X-100 and 0.15 μg proteinase K (Roche)] at 55°C. The following day, samples were heat inactivated for 5′ at 95°C and centrifuged to remove debris. Next, 1 μl of supernatant was used as template DNA in PCR. Templates were checked for presence of rtTA and/or TRE. The following PCR mix was used: 10× FastStart DNA Polymerase buffer with MgCl_2_ (Roche), 25 mM dNTPs, primers (10 mM), FastStart DNA polymerase (5 U/μl; Roche) and sterilized water. The PCR program consisted of 4′ denaturation at 94°C, followed by 30 cycles of amplification through 30′′ at 94°C, 30′ at 60°C, and 90′′ at 72°C, and ended with 5′ at 72°C. PCR products were visualized by adding 10 μl 3× loading mix [30% Orange G (Sigma), 0.2% GelRed (Biotium) in H_2_O] to 5 μl of PCR product and separating using gel electrophoresis on a 1.5% agarose gel. Gels were scanned using Gel Doc XR+ (Bio-Rad) Molecular Imager with Image Lab software. The TRE transgene was amplified using forward primer 5′-GCTTAGATCTCTCGAGTTTAC-3′ and reverse primer 5′-ATGGAGGTCAAAACAGCGTG-3′. The rtTA transgene was amplified using forward primer 5′-CAGCAGGCAGCATATCAAGGT-3′ and reverse primer 5′-GCCGTGGGCCACTTTACAC-3′.

### Repeat Length PCR

Repeat length was determined according to an in-house PCR protocol. Brain tissue from mice having 11CGGs (positive control), wildtype mice (negative control) and TRE-103CGG-GFP 4 weeks old mice were incubated overnight in 300 μl tail mix buffer [50 mM Tris pH = 7.5, 10 mM EDTA, 150 mM NaCl, 1% SDS and 20 μl proteinase K (10 mg/ml; Roche Cat. #3115852)] at 55°C. The next day, 100 μl 6 M NaCl was added to the samples and samples were shaken very well to induce precipitation of cell debris. Samples were centrifuged (10,000 *g* at RT for 10 min) to remove cell debris. The supernatant was transferred to a new tube and 1 ml 100% EtOH was added (shake very well). Tubes were centrifuged at 10,000 *g* for 10 min to form DNA pellet. Next, the supernatant was discarded and DNA pellet was washed with 500 μl 70% EtOH. Samples were centrifuged at 10,000 *g* for 5–10 min. The supernatant with EtOH was discarded and the DNA pellet was left to dry to the air for a couple of minutes. The DNA pellet was resuspended in 100 μl sterilized water. Next, 1 μl of supernatant was used as template DNA in the PCR reaction mix (total volume 21 μl). Following PCR mix was used: 10 μl Betaine (5 M), 4 μl 5× expand HF buffer without Mg^2+^, 1.5 μl MgCl_2_ (25 mM) 1 μl forward primer (10 μM), 1 μl reverse primer (10 μM), 0.2 μl dNTP mix (100 mM) (25 mM each), 0.2 μl FastStart Taq DNA polymerase (5 U/μl; Roche) and 2.1 μl sterilized water. The PCR program consisted of 10’ denaturation at 98°C, followed by 35 cycles of amplification through 35 s at 98°C, 35 s at 58°C and 3 min at 72°C, and ended with a cooling step at 15°C. For quantification of the DNA size, 1 μl 1 Kb Plus DNA ladder (Thermo Fisher Scientific; Cat. # 10787018) was used with and without 0.2% GelRed (Biotium) in dH_2_O. Staining with GelRed after electrophoresis run is necessary because GelRed interferes with the DNA and therefore influences CGG repeat measurement. To front track DNA separation during gel electrophoresis, 10 μl 30% Orange G (Sigma) loading dye was added to 5 μl of PCR product on the 1.5% agarose gel. After gel electrophoresis run, the agarose gel was stained for 30 min in 500 ml 1X TBE-buffer (1L 5X TBE-buffer: 54 g Tris (CAS #77-86-1), 27.5 g boric acid (CAS #10043-35-3) and 20 ml 0.5 M EDTA pH = 8.0 (CAS #60-00-4) + 50 μl 0.2% GelRed (Biotium). Gels were scanned using Gel Doc XR+ (Bio-Rad) Molecular Imager with Image Lab software. The CGG repeat was amplified using the following forward primer 5’-ATCCACGCTGTTTTGACCTC-3’ and reverse primer 5’-CCAGTGCCTCACGACCAAC-3’.

### RNA Isolation and cDNA Synthesis

RNA isolation was performed on dox and sucrose treated 16 weeks old *CamKII-*α-rtTA/TRE-103CGG-GFP mice. Per treatment group *n* = 3 brains were used for RNA isolation. Prior to lysing, samples were thawed on ice and supplied with RIPA-buffer containing 0.05% protease inhibitors (Roche), 0.3% 1 M DTT (Invitrogen) and 40U RNase Out (Roche). Samples were mechanically lysed, followed by 30 min of incubation on ice. After 30 min of incubation, mechanical lysing was repeated to ensure total homogenization. Homogenate was added to RNA Bee (Tel-Test) in a 1:10 (v/v) ratio and mixed thoroughly. Chloroform (Millipore) was added to mixture in a 1:5 ratio (v/v), mixed thoroughly and incubated on ice for 15 min. After incubation the mixture was centrifuged for 15 min at 4°C and supernatant was collected and supplied with 0.6× (v/v) 100% 2-propanol (Honeywell). After 15 min centrifugation at 4°C, supernatant was discarded. Remaining pellet was washed with 80% EtOH (Honeywell) in duplicate with brief centrifugation at 4°C between washes. Following removal of residual supernatant, 50 μl dH_2_O was added and concentration was determined using the NanoDrop 2000 (Thermo Fisher Scientific).

### Quantitative Real-Time PCR

Reverse transcriptase (RT) was performed using 1 μg of RNA with the iScript cDNA synthesis kit (Biorad) according to manufacturer’s instructions. RNA was treated with DNase before cDNA synthesis. Q-PCR using iTaq Supermix (BioRad) was performed on 0.1 μl RT product. Cycling conditions were an initial denaturation of 3 min at 95°C, followed by 35 cycles of each 5 s at 95°C and 30 s at 60°C. As a reference gene GAPDH was used. For statistical analysis the two-sample unpaired *t*-test assuming equal variance was used.

### Immunohistochemical Staining

Tissues were fixed overnight in 4% paraformaldehyde (PFA) at 4°C and embedded in paraffin according to in-house protocols. Sections of 6 μm were cut and placed on silane coated slides (Klinipath). The sections were deparaffinized in decreasing concentrations of alcohol—starting with xylene and ending in demineralized H_2_O—before performing antigen retrieval by microwave treatment in 0.01 M sodium citrate (pH = 6). Endogenous peroxidase activity was blocked with 0.6% H_2_O_2_ in PBS. When staining for FMRpolyG an additional incubation with proteinase K (5 μg/ml) was performed for 20–30 min at 37°C to ensure optimal antibody binding. Staining was performed overnight at 4°C with primary antibodies diluted in PBS/0.5% milk/0.15% glycine (PBS+). Staining with secondary antibodies was performed at RT for 60 min. Antigen-antibody complexed were visualized using DAB-substrate (DAKO), after which slides were counterstained with hematoxylin for 5 min and subsequently mounted with Entellan (Merck Milipore International). Antibodies used are listed in Table 1 hereafter.

**TABLE 1:** Antibodies.

**Target**	**Dilution**	**Host**	**Source**	**Catalog No.**
FMRpolyG (8FM)	1:10	Mouse	Gift from N. Charlet-Berguerand, IGBMC	X
GFP	1:2000	Mouse	Roche	11814460 001

*Mouse specific anti-GFP and anti-FMRpolyG (8FM) antibodies were used to visualize GFP expression and the FMRpolyG protein aggregates in mouse brain, respectively.*

### Behavioral Testing

Muscle function was tested using a hanging wire test. A metal wire with a diameter of 2 mm was suspended around 20 cm above a cage. The mouse was brought to the wire so that he could grasp the wire with his front paws after which the latency to fall was recorded. The maximal trial duration was 60 s. In addition, we used the Bioseb grip strength test (Bioseb, Vitrolles, France). For this test, the mouse was placed on a metal grid and after he clamped to the grid with all four limbs, he was gently pulled down by the base of his tail. The maximal force was measured and the average of three consecutive trials was calculated.

The fine motor coordination of the mice was tested on the balance beam. During 2 consecutive days, the mice were habituated to the setup that consisted of a horizontal wooden beam with a diameter of 12 mm and a length of 100 cm located approximately 50 cm above a table. Each mouse was placed on one side of the beam and walked over the beam to a home cage at the other side of the beam. After two trials, the beam was replaced by one with a diameter of 8 mm and also on this beam two trials were performed. On the third day, the performance of the mice was quantified by counting the number foot slips and falls. Each mouse crossed each beam twice and the average time required to reach the other side of the beam was measured, taking only trials without falls into account.

Locomotor patterns were recorded on a horizontal ladder flanked by two plexiglass walls spaced 2 cm apart (ErasmusLadder, Noldus, Wageningen, Netherlands) as described previously (Vinueza Veloz et al., 2015). The ladder consisted of two rows of 37 rungs placed in an alternated high/low pattern. The rungs were spaced 15 mm apart and the height difference between high and low rungs was 9 mm. Each rung was connected to a pressure sensor recording rung touch. During a trial, the mouse had to walk from a shelter box on one side of the ladder to another on the other end. Trial start was indicated by lighting an LED in the shelter box followed 3 s later by a strong tail wind. Early escapes, thus before the LED was switched on, were discouraged as they triggered a strong head wind. In between trials, there was a resting period. Mice were first habituated to the setup by letting them freely explore the ladder for 15 min during which no light or air cues were given. On the next day, training started with 44 trials on each day. The initial training consisted of six daily sessions, after which the mice were measured once a week. Sensor touches were filtered to delete single backsteps or fake hind limb steps using the factory settings. For the further analysis, we used the touches of the front limbs with the first and the last step of each trial being deleted.

Using the water maze test, we quantified the spatial memory of the mice. Each mouse was placed on the border of a circular pool with a diameter of 120 cm filled with a mixture of water and non-toxic white paint kept constant at 26°C. In the pool, a platform with a diameter of 11 cm diameter was hidden 1 cm below the water surface. The time to find the platform was recorded on two trials each day on 5 consecutive days. When the mouse did not find the platform within 60 s, the trial was stopped. On days 6 and 7, a probe trial was given. During the probe trials, the platform was absent and the mice were allowed to swim for 60 s while their trajectory was tracked (EthoVision XT11, Noldus, Wageningen, Netherlands). The data of the probe trials were analyzed by subdividing the pool in four quadrants, with the original position of the platform in the middle of quadrant 3. We marked the original platform position as well as the same shape at the corresponding position in the other three quadrants and counted how often the mouse passed the borders of each of these positions per trial. We considered a crossing if it involved more than 50% of the body of the mouse. On top of that, we also quantified the time spent in each quadrant.

The battery of behavioral tests is schematically represented in time in Figure 1B.

### Statistics

Behavioral performance on each paradigm was compared between mice treated with and without doxycycline. The statistical tests used are mentioned in the Results section, whereby we used non-parametric tests for data that were not normally distributed. Throughout the manuscript, we considered a *p-*value of 0.05 or less as indication for statistical significance.

## Results

### Expanded CGG Expression Results in Inclusion Formation in the Hippocampus and Basal Ganglia

**FIGURE 2:**
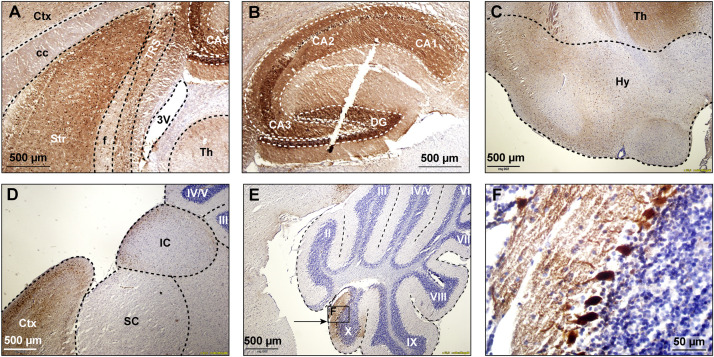
GFP expression in multiple brain regions. GFP expression (brown staining) was visualized using immunohistochemical staining with a mouse specific anti-GFP antibody in sagittal brain sections at 12 weeks after onset of dox-treatment. Strong expression of GFP was present in the striatum **(A)**, the hippocampus **(B)**, the hypothalamus **(C)**, and the cerebral cortex **(D)**. Lower levels of expression were present in the superior and inferior colliculus **(D)**. In the cerebellum, GFP expression was only observed in vermal lobule X (**E**, indicated area amplified in **F**). 3V, third ventricle; cc, corpus callosum, Ctx, cerebral cortex; DG, dentate gyrus; f, fornix; Hy, hypothalamus; IC, inferior colliculus; SC, superior colliculus; Str, striatum; Th, thalamus; TRS, triangular nucleus of the septum.

First of all, we studied the expression pattern of the FMRpolyG-GFP fusion protein in *CamKII-*α-rtTA/TRE-103CGG-GFP mice after induction of transgene expression by the addition of doxycycline (dox) to the drinking water. First, repeat length in the transgene was verified using an in-house PCR protocol. Repeat length PCR shows the repeat size of 103× CGGs at approximately 480 bp compared to the control 11 CGGs length at 290 bp (Supplementary Figure S1A). To verify whether dox treatment did not affect murine *Fmr1* mRNA expression, we performed quantitative real-time PCR on brain tissue of treated and control mice. The data show that dox treatment had no effect on *Fmr1* mRNA expression in the brain as tested in the hippocampus (Supplementary Figure S1B). Since the transgene expression was under the *CamKII-*α promoter, we expected the FMRpolyG protein to be present in neurons of, among other regions, the hippocampus, the neocortex, the basal ganglia, and in the posterior part of the cerebellum, more specifically lobule X (Hasegawa et al., 2009; Wang et al., 2013). In our hands, already after 4 weeks of dox treatment, GFP expression, indicative of FMRpolyG expression, was found in all aforementioned brain regions. After 12 weeks of dox treatment, the expanded CGG repeat was strongly expressed in the striatum of the basal ganglia, the hippocampus, the neocortex, and lobule X of the cerebellum (Figures 2A,B,D,E). Low to modest expression of GFP was present at 12 weeks in the hypothalamus, the colliculus inferior and superior (Figures 2C,D), and other sub-regions of the midbrain.

**FIGURE 3:**
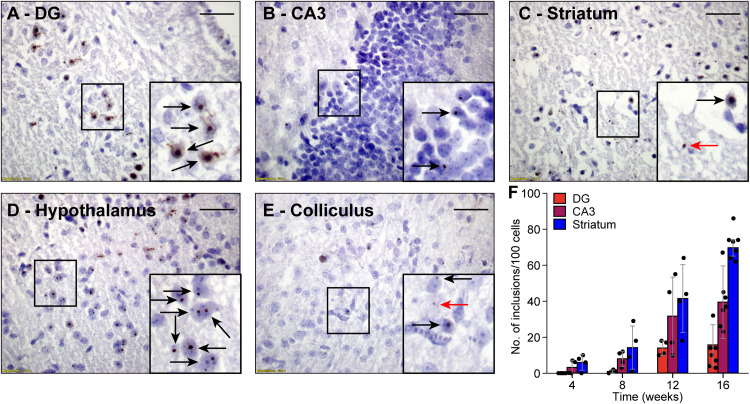
FMRpolyG-positive inclusions are predominantly located in the nucleus. FMRpolyG-positive inclusions, visible as black dots, were stained using the mouse anti-FMRpolyG (8FM) antibody. Most often, the FMRpolyG-positive inclusions were observed in the nuclei (black arrows) but occasionally also outside the nuclei (red arrows). FMRpolyG-positive inclusions were found in the dentate gyrus (DG, **A**) and the CA3 region of the hippocampus **(B)**, the striatum **(C)**, the hypothalamus **(D)**, and in the colliculi **(E)**. Rectangles indicate areas enlarged in insets. Scale bars = 50 μm. **(F)** The prevalence of FMRpolyG-positive inclusions increased over time after onset of dox treatment. Bars indicated average values and error bars the sd.

Next, we investigated whether FMRpolyG expression was associated with the formation of nuclear inclusions in the *CamKII-*α-rtTA/TRE-103CGG-GFP mice. To this end, we compared brain sections stained for FMRpolyG from mice that did receive dox with those from mice that did not. As expected, we could not detect any inclusions in the control mice. However, the mice treated with dox developed spherical FMRpolyG-positive inclusions in most of the brain regions in which GFP expression was observed. The highest density of inclusions was found in the striatum, the CA3 region of the hippocampus and the hypothalamus (Figures 3B–D). Lower densities were present in the DG region of the hippocampus, as well as in the inferior and the superior colliculus (Figures 3A,E). We did not observe a perfect correlation between GFP expression and the occurrence of inclusions: in lobule X of the cerebellum, no inclusions were found despite the presence of GFP (Figures 2E,F). In general, during 12 weeks of dox treatment, the number of inclusions increased over time with regional differences. Quantification of FMRpolyG-positive inclusions (Figure 3F) was only done in the hippocampus and the striatum of the basal ganglia, since these regions are known to be involved in FXTAS disease pathology (Greco et al., 2002). Irrespective of the brain region involved, most inclusions were located intranuclearly. Sometimes two or more smaller inclusions were located in the same nucleus. In summary, dox induced the production of CGG RNA in *CamKII-*α-rtTA/TRE-103CGG-GFP mice in several brain regions, which resulted in the formation of FMRpolyG-positive nuclear inclusions, predominantly in the striatum and the hippocampal CA3 region.

### Absence of Behavioral Phenotype in Mice Expressing FMRpolyG-Positive Inclusions

**FIGURE 4:**
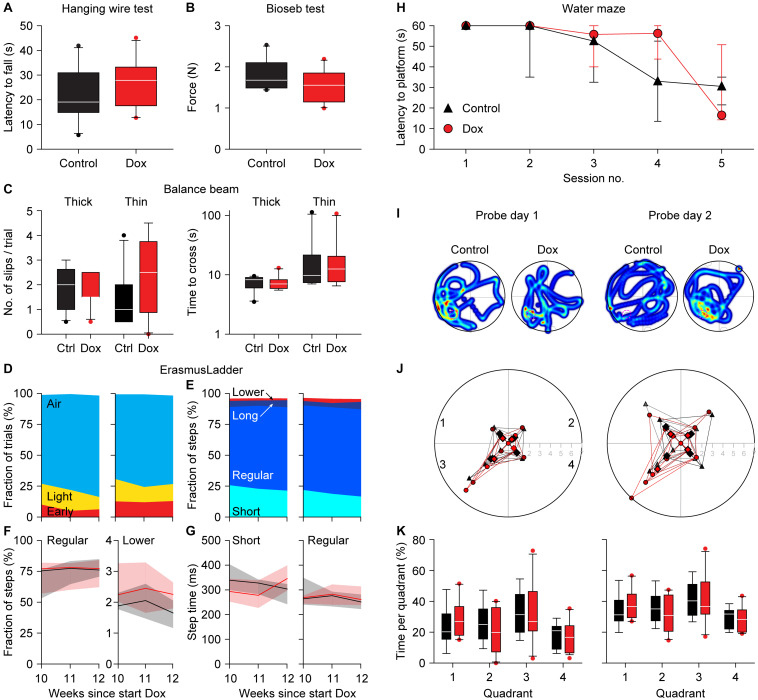
Absence of a clear behavioral phenotype in dox-treated mice. Neither the hanging wire test **(A)** nor the Bioseb grip strength test **(B)** demonstrated an impact of dox-treatment on muscle strength. **(C)** Also, the balance beam test failed to find consistent differences in either the number of slips (left) or the time to cross (right). The tests were performed on a thick (12 mm diameter) and a thin (8 mm diameter) wooden beam. **(D)** On the ErasmusLadder, trial starts were indicated by lighting an LED in the start box, followed 3 s later by a strong tail wind. The fraction of trials with starts before the visual cue (“Early”), during the visual cue (“Light”) or after the start of the tail wind (“Air”) were comparable between control and dox-treated mice. Data recorded at 10, 11, and 12 weeks after the start of dox-treatment. **(E)** The ErasmusLadder consists of alternating high and low rungs. The fractions of short steps (from one high rung to the next), regular steps (from one high rung to the second high rung), long steps (from on high rung to another, skipping at least two) and lower rung touches were also similar for both groups, as further illustrated for the fraction of regular steps and lower rung touches **(F)** as well as for step times **(G)**. The data in **(F,G)** show the medians with the shades indicating the inter-quartile range. See Table 2 for a more extensive statistical analysis of the ErasmusLadder test. **(H)** In the water maze, the mice had to find a platform hidden just below the water surface. As the water was made opaque, the mice could not see the platform. During 5 consecutive training days, the latency to find the platform decreased both in control and dox-treated mice. **(I)** On the next 2 days, the hidden platform was removed (probe trials) and the trajectories of the mice were recorded. The heat maps indicate the time spent per area of two exemplary mice. The original location of the hidden platform is indicated by a pink dashed circle in quadrant 3. **(J)** On the first probe day, the mice crossed the location where the hidden platform had been more often than the analogous regions of the other quadrants. On the second day, the control mice no longer searched more often in the area where the hidden platform had been (*p* = 0.624, Mann-Whitney test), while the dox-treated mice kept searching specifically around the location where the hidden platform had been (*p* = 0.005, Mann-Whitney test). **(K)** This retention, however, was not noticeable when comparing the times spent per quadrant. Unless indicated otherwise, behavioral tests were performed during the 12^th^ week of dox-treatment. Group sizes were 10 mice.

To test whether the expression of the CGG repeat and the resulting nuclear inclusions had any impact on mouse behavior, we subjected the mice to a battery of behavioral tests. To control for possible confounding problems with the general condition of the mice, we first tested the muscle strength using the hanging wire and the Bioseb grip strength tests 12 weeks after the start of the dox treatment. The latency to fall was 22.0 ± 11.0 vs. 26.5 ± 10.2 s (control vs. dox mice, averages ± s.d., *p* = 0.385, *t* = 0.944, *df* = 17, *t-*test Figure 4A) during the hanging wire test and the force was 1.79 ± 0.37 vs. 1.53 ± 0.39 N (control vs. dox mice, averages ± s.d., *p* = 0.336, *t* = 0.991, *df* = 17, *t*-test Figure 4B) during the Bioseb grip strength test. We therefore conclude that there were no indications for changes in muscle strength due to the dox treatment.

Next, we tested the overall motor control and balance on the balance beam after 12 weeks of dox treatment. The numbers of hind foot slips per trial were comparable between control and dox mice [thick beam: 2.0 (inter-quartile range (IQR): 1.5) vs. 1.5 (IQR: 0.9), *p* = 0.876, *U* = 47.5; thin beam: 1.0 (IQR: 1.4) vs. 2.5 (IQR: 2.5), *p* = 0.220, *U* = 33.5, medians, Mann-Whitney tests, Figure 4C]. Also the time required to cross the beam were not really different between control and dox mice [thick beam: 8.3 (IQR: 2.5) vs. 7.0 (IQR: 1.9) s, *p* = 0.593, *U* = 42.5; thin beam: 9.8 (IQR: 8.6) vs. 12.5 (IQR: 9.1), *p* = 0.820, *U* = 46.5, medians, Mann-Whitney tests, Figure 4C]. We take this as a sign that the treatment did not impair the overall motor control and ability to keep balance.

We continued by describing the behavior on the ErasmusLadder, which is a horizontal ladder consisting of two rows of rungs in an alternating high/low pattern spanning the space between two shelter boxes. After habituation and initial training, we measured the performance at 10, 11, and 12 weeks after the start of dox treatment. The start of each trial was indicated by switching on an LED in the start box and this was followed by a strong tail wind 3 s later. In roughly 75% of the trials, the mice waited until the tail wind started before leaving the start box. Leaving upon perception of the visual cue or even before that was observed less often. Changes in this pattern could be a sign of cognitive impairment (Vinueza Veloz et al., 2012), but these were not observed between control and dox mice (*p* = 0.516, 3 × 2 Fisher’s exact test, Figure 4D).

Next, we characterized the stepping pattern on the ErasmusLadder. Wild type C57BL/6J mice have a tendency to avoid the lower rungs and typically make steps from one high rung to the next or the second next high rung (Vinueza Veloz et al., 2015). We considered these small and regular steps, respectively. Long steps, skipping at least two higher rungs, and lower rung steps occurred much less often, as did other irregular steps such as backwards walking. Thus, also regarding the stepping pattern, no impact of the dox treatment was observed (Table 2 and Figures 4E–G).

**TABLE 2:** Step size statistics for the ErasmusLadder.

	**Treatment**	**Median**	**IQR**	***p***	**F**	**df**	**Test**
**Step lengths (%)**							
Short steps (step size = 2)	-Dox	6.4	15.8	0.687	0.168	1	Repeated measures ANOVA
	+Dox	7.1	22.8				
Regular steps (step size = 4)	-Dox	76.1	14.2	0.699	0.154	1	Repeated measures ANOVA
	+Dox	77.2	22.0				
Long steps (step size ≥ 6)	-Dox	3.4	7.9	0.688	0.166	1	Repeated measures ANOVA
	+Dox	5.1	4.1				
Lower rung steps	-Dox	1.7	0.9	0.153	2.226	1	Repeated measures ANOVA
	+Dox	2.3	0.8				
Backsteps	-Dox	1.3	1.0	0.629	0.241	1	Repeated measures ANOVA
	+Dox	2.1	1.8				
**Step times (ms)**							
Short steps (step size = 2)	-Dox	303	82	0.751	0.104	1	Repeated measures ANOVA
	+Dox	346	101				
Regular steps (step size = 4)	-Dox	251	59	0.588	0.304	1	Repeated measures ANOVA
	+Dox	261	75				

*The percentages of steps to higher rungs, being either short, regular or long, as well as those to lower steps (irrespective of stride length), and backward steps were compared at 10, 11, and 12 weeks after onset of dox treatment. Note that the percentages do not add to 100% as some irregular types of steps were not considered here (in particular, steps starting from lower rungs). Of the two most frequent step categories, also the step times are indicated and compared. The values were first calculated per mouse, and then compared between the two groups (n = 10 mice/group). The median and interquartile range (IQR) values in this table refer to the recording session at 12 weeks after onset of dox treatment. All values refer to front paw movements. p-values reflect the between-subject comparisons of repeated measures ANOVAs. Since not a single p-value was close to the threshold for significance, no correction for multiple comparisons was applied.*

Finally, to test for putative defects in spatial memory formation, we subjected the mice to the Morris water maze test around 12 weeks after the start of the dox treatment. During 5 consecutive days, the mice were trained to find a hidden platform just below the surface of an opaque, circular pool. Over the sessions, both control and dox-treated mice managed to be faster in finding the hidden platform, with no statistically significant differences between the two groups [*p* = 0.134, *F*_(1, 17)_ = 2.479, repeated measures ANOVA, Figure 4H]. On the next 2 days, the experiment was repeated—but without a hidden platform. On these probe trials we made video recordings of the mice (Figure 4I). First, we counted how often the mice crossed the location where the hidden platform had been during the training sessions and compared these with crosses of the analogous locations in the other three quadrants. During the first probe trial, both control and dox treated mice had a preference for the real location (in quadrant 3) over the other areas (control: 2.2 ± 1.5 crosses per trial of the real location vs. 1.0 ± 0.9 crosses of the other locations, *p* = 0.021, *U* = 60.5, Mann-Whitney test, dox mice 2.0 ± 2.2 vs. 0.6 ± 0.7 crosses, averaged ± ss, *p* = 0.107, *U* = 101.5, Mann-Whitney test, control vs. dox mice: *p* = 0.813, χ^2^ = 0.95, χ^2^ test). During the second probe trial, the preference of the control mice for the real location was gone (1.8 ± 1.2 vs. 1.6 ± 1.3 crosses, *p* = 0.624, *U* = 108.0, Mann-Whitney test), but remained present in the dox treated mice (3.3 ± 2.3 vs. 1.1 ± 1.1 crosses per trial, *p* = 0.005, *U* = 63.0, Mann-Whitney test). This difference between control and dox mice was on the border of statistical significance (*p* = 0.061, χ^2^ = 7.36, χ^2^-test, Figure 4J). This might indicate that the dox-treated mice had more trouble understanding that the hidden platform was no longer in place. This, however, was not reflected in the relative dwell times per quadrant [*p* = 1.00, *F*_(1, 17)_ = 0.000, repeated measures ANOVA, Figure 4K], which leads us to conclude that also the Morris water maze did not reveal convincing differences in behavior due to activation of the premutation.

## Discussion

Wide-spread occurrence of nuclear inclusions is a major hallmark of FXTAS. To date, it is a matter of debate whether these inclusions contribute to cellular pathology in FXTAS, or—in contrast—slow down the disease process by sequestering toxic RNA and proteins. Such a protective function has been suggested for FXTAS (Greco et al., 2006; Hagerman and Hagerman, 2016), but also for other protein-aggregation disorders, such as Huntington’s disease and SCA1 (Klement et al., 1998; Saudou et al., 1998; Cummings et al., 1999; Arrasate et al., 2004). To study the relation between the development of intranuclear inclusions and behavioral deficits, we used a novel, inducible and neuron-specific mouse model for FXTAS under the control of the *CamKII-*α promoter. Expression of an expanded 103CGG repeat RNA transgene is induced by dox and is under the control of the Tet-On system. This inducible mouse model shows no evidence of expression in the absence of dox (i.e., no leakage of expression), and was induced after completion of normal development to avoid interaction with developmental processes. Within a month after transgene induction, FMRpolyG-positive nuclear inclusions were found in the striatum and the CA3 region of the hippocampus. Two months after the occurrence of the first nuclear inclusions, the inclusions were abundant in most brain areas in which the *CamKII-*α promoter is active such as the hippocampus, neocortex and the striatum. Yet, we could not identify a robust behavioral phenotype that could be caused by the inclusion pathology in these mice. Several mouse models have significantly contributed to our understanding of the molecular mechanisms underlying FXTAS and have characterized disease progression. Previously, we found in a different inducible mouse model for FXTAS, using the heterogeneous nuclear ribonucleoproteins (*hnRNP*) promoter, a rapid death after dox-induction. The neuronal level of transgene expression in these mice was low, and nuclear inclusions were sparse or even absent in the brain (Hukema et al., 2014). In contrast, in a third mouse line, under control of the brain-specific protease-resistant-protein (*PrP*) promoter, we observed both the formation of nuclear inclusions and behavioral deficits (Hukema et al., 2015). These mice developed only a deficit in the compensatory eye movement pathway after 20 weeks of treatment with dox. Although expression of the transgene containing the expanded CGG repeat mRNA was found in the hippocampus, lobule X of the cerebellum and the striatum, these expression levels were low with the exception of lobule X of the cerebellum where expression was the most profound. Together, these results lead us to question whether the development of nuclear inclusions is indeed the cause of developing FXTAS symptoms. Therefore, we developed a new inducible transgenic mouse model under the control of the *CamKII-*α promoter expecting stronger expression in the brain.

In our *CamKII-*α-rtTA/TRE-103CGG-GFP mouse model, the expression of GFP followed that of the previously described distribution of the *CamKII-*α promoter (Wang et al., 2013). Immunohistochemical staining shows the strongest GFP expression in the striatum, the CA3 region of the hippocampus and lobule X of the cerebellum. Moderate GFP expression was found in the neocortex, the dentate gyrus, the hypothalamus and several midbrain areas. In all of these regions, with the notable exception of the cerebellum, also nuclear inclusions were formed. If nuclear inclusions in these areas would result in functional deficits, a broad range of behavioral impairments is to be expected. As a consequence, typical cerebellar symptoms, although prominent in FXTAS patients (Hagerman et al., 2001; Tassone et al., 2007; Hagerman and Hagerman, 2013, 2016), were not expected in our mouse model since the *CamKII-*α is only expressed in a very limited part of the cerebellum. We therefore focused on spatial learning, that has previously been shown to be affected in a knock-in mouse model (“the Dutch mouse”) (Van Dam et al., 2005; Hunsaker et al., 2009), and striatal motor coordination functions, as they also occur as parkinsonism in patients (Hagerman et al., 2001).

An intact hippocampus is essential for normal spatial learning in the water maze (D’Hooge and De Deyn, 2001; Okada and Okaichi, 2009; Laeremans et al., 2015). Our mice showed no, or only marginal, deficits at the water maze test, arguing against a severely impaired hippocampal function. The striatum is vital for motor control and striatal damage leads to impaired behavior on the balance beam (Shear et al., 1998; Feng et al., 2014), which was not observed in our mice. This lack of an effect on motor coordination was further substantiated by equal performance of treated and control mice on the ErasmusLadder and the grip tests. Although we cannot exclude that there were subtle behavioral deficits that we did not observe, it is safe to state that there were no major changes in behavioral performance in spite of the abundance of nuclear inclusion in the dox treated mice.

The expanded CGG RNA and proteins can aggregate with many other molecules into nuclear inclusions (Ma et al., 2019). The expanded CGG RNA on itself is not enough to induce toxicity and that the production of an out-of-frame FMRpolyG protein due to RAN translation is necessary for cellular toxicity (Galloway and Nelson, 2009; Hashem et al., 2009; Sellier et al., 2017; Derbis et al., 2018). Our present results indicate that the development of FMRpolyG-positive nuclear inclusions themselves are probably not very detrimental to the function of neurons. It remains to be seen whether aggregation is an active process, aimed at sequestering toxic molecules and thereby slowing down the disease progression, or more an epiphenomenon that is a physical consequence of the molecular structure of the expanded CGG RNA and/or RAN translation protein FMRpolyG.

## Data Availability Statement

The raw data supporting the conclusions of this article will be made available by the authors, without undue reservation, to any qualified researcher.

## Ethics Statement

All experiments involving mice were performed according to Dutch law and following institutional guidelines (Erasmus MC, Rotterdam, Netherlands) in confirmation with EU directive 2010/63. Prior to the start of the experiments, project licenses were obtained from the national authority (Centrale Commissie Dierproeven, The Hague, Netherlands) after review by an independent ethical committee (DEC Consult, Soest, Netherlands) and filed under numbers AVD101002015290 and AVD1010020197846.

## Author Contributions

SH, RH, and LB conceived the project. SH, RM, ET, VB, and RV performed the experiments. SH, RM, RW, RH, and LB analyzed the data. SH, RW, and LB wrote the manuscript with input from all authors.

## Conflict of Interest

The authors declare that the research was conducted in the absence of any commercial or financial relationships that could be construed as a potential conflict of interest.

## Supplementary Material

Supplementary Figure 1 CGG repeat length verification and *Fmr1* mRNA expression in dox-induced transgenic mice. **(A)** Gel electrophoresis analysis shows that the CGG repeat length in the *CamKII-*α-rtTA/TRE-103CGG-GFP transgenic mice is in the premutation range. Repeat length PCR shows the repeat size of 103× CGGs and 11× CGGs at approximately 480 and 290 bp, respectively. +L = Ladder with GelRed; -L = Ladder without GelRed; MUT = TRE-103CGG-GFP (mouse ID: 20464-1); 11CGG = normal repeat length (positive control); WT = wildtype (negative control); H_2_O = water control. **(B)** Quantification of *Fmr1* mRNA expression in the hippocampus of dox-induced (*n* = 3) and control (sucrose; *n* = 2) mice 16 weeks after start of the dox treatment. Dox-induction does not affect *Fmr1* mRNA expression levels in these transgenic mice. Measurements were repeated twice with the same conditions using q-RT-PCR and normalized to the average level found in the control-dox mice.
