# Supplementary figures and images for: Lack of a Clear Behavioral Phenotype in an Inducible FXTAS Mouse Model Despite the Presence of Neuronal FMRpolyG-Positive Aggregates
Source: Front Mol Biosci. 2020 Dec 14;7:599101. doi: 10.3389/fmolb.2020.599101 (PMC7768028; doi:10.3389/fmolb.2020.599101)

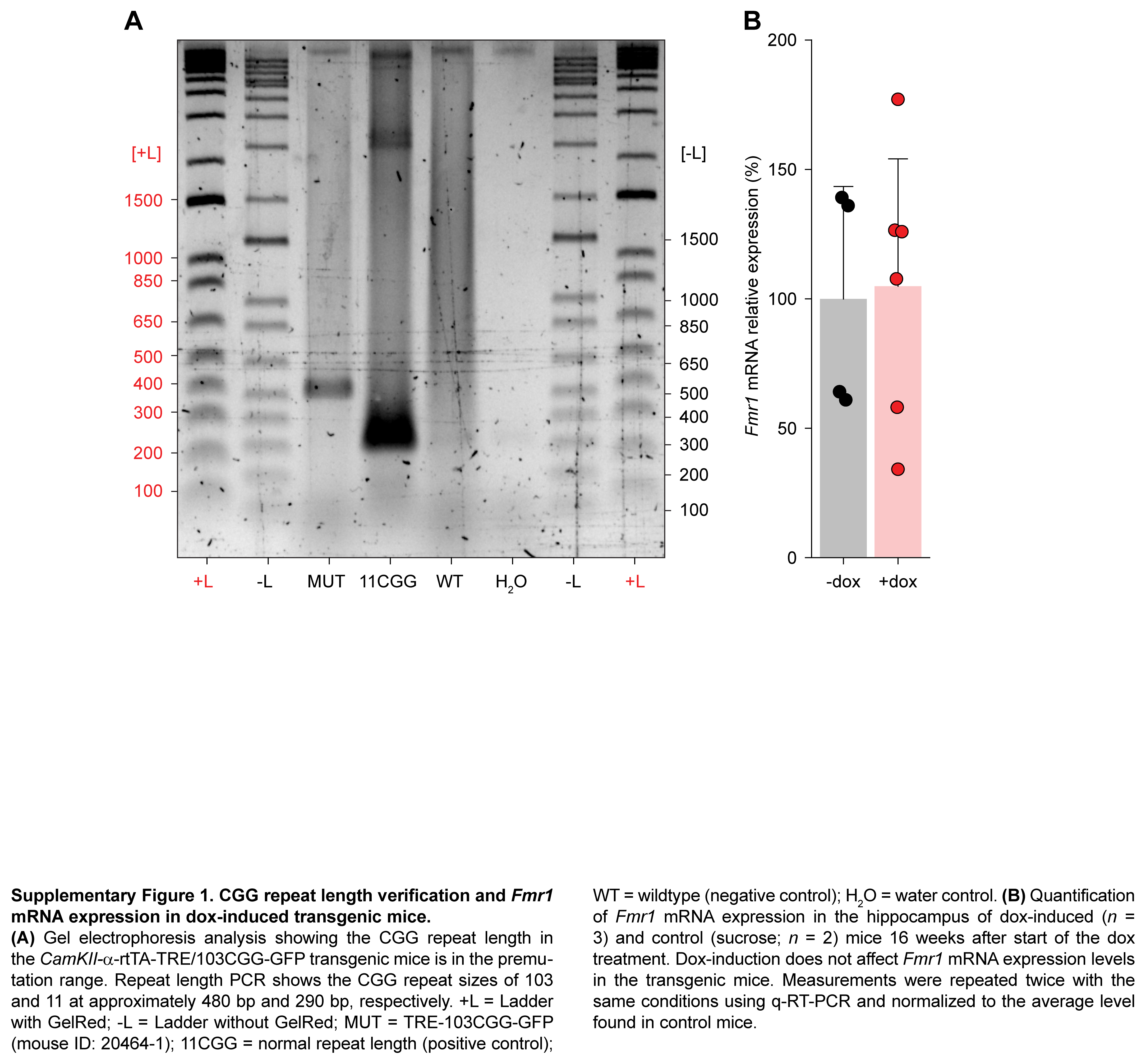

Supplement: Supplementary Figure 1 — CGG repeat length verification and Fmr1 mRNA expression in dox-induced transgenic mice. (A) Gel electrophoresis analysis shows that the CGG repeat length in the CamKII-α-rtTA/TRE-103CGG-GFP transgenic mice is in the premutation range. Repeat length PCR shows the repeat size of 103× CGGs and 11× CGGs at approximately 480 and 290 bp, respectively. +L = Ladder with GelRed; -L = Ladder without GelRed; MUT = TRE-103CGG-GFP (mouse ID: 20464-1); 11CGG = normal repeat length (positive control); WT = wildtype (negative control); H2O = water control. (B) Quantification of Fmr1 mRNA expression in the hippocampus of dox-induced (n = 3) and control (sucrose; n = 2) mice 16 weeks after start of the dox treatment. Dox-induction does not affect Fmr1 mRNA expression levels in these transgenic mice. Measurements were repeated twice with the same conditions using q-RT-PCR and normalized to the average level found in the control-dox mice. [file Image_1.TIF]
